# A SPR Aptasensor for Detection of Avian Influenza Virus H5N1

**DOI:** 10.3390/s120912506

**Published:** 2012-09-13

**Authors:** Hua Bai, Ronghui Wang, Billy Hargis, Huaguang Lu, Yanbin Li

**Affiliations:** 1 Department of Biological and Agricultural Engineering, University of Arkansas, Fayetteville, AR 72701, USA; E-Mails: bhua@uark.edu (H.B.); rwang@uark.edu (R.W.); 2 Department of Poultry Science, University of Arkansas, Fayetteville, AR 72701, USA; E-Mail: bhargis@uark.edu; 3 Animal Diagnostic Laboratory, Pennsylvania State University, University Park, PA 16802, USA; E-Mail: hx115@psu.edu

**Keywords:** aptamer, surface plasmon resonance, biosensor, avian influenza virus

## Abstract

Rapid and specific detection of avian influenza virus (AIV) is urgently needed due to the concerns over the potential outbreaks of highly pathogenic H5N1 influenza in animals and humans. Aptamers are artificial oligonucleic acids that can bind specific target molecules, and show comparable affinity for target viruses and better thermal stability than monoclonal antibodies. The objective of this research was to use a DNA-aptamer as the specific recognition element in a portable Surface Plasmon Resonance (SPR) biosensor for rapid detection of AIV H5N1 in poultry swab samples. A SPR biosensor was fabricated using selected aptamers that were biotinylated and then immobilized on the sensor gold surface coated with streptavidin via streptavidin-biotin binding. The immobilized aptamers captured AIV H5N1 in a sample solution, which caused an increase in the refraction index (RI). After optimizing the streptavidin and aptamer parameters, the results showed that the RI value was linearly related (R^2^ = 0.99) to the concentration of AIV in the range of 0.128 to 1.28 HAU. Negligible signal (<4% of H5N1) was observed from six non-target AIV subtypes. The AIV H5N1 in poultry swab samples with concentrations of 0.128 to 12.8 HAU could be detected using this aptasensor in 1.5 h.

## 1. Introduction

Avian influenza (AI), also called “bird flu”, is a well-known pathogenic menace in the poultry industry caused by type A influenza virus, causing huge economic losses around the World [1,2]. The continued presence of high-pathogenicity avian influenza (HPAI) H5N1 in birds has been reported in Asia, Africa, and Europe [3]. Generally, humans are not infected by avian influenza virus, but the first human cases of AIV H5N1 were reported in Hong Kong in 1997 [4,5], which caused eighteen infections and six deaths. Since 2003, there has been a total of 603 confirmed human cases and 356 deaths reported by the WHO [6].

The recent increasing emergence of infectious influenza diseases has prompted interest in the detection of AIV H5N1 in humans as well as animals. A variety of technologies for diagnosing AIV infection have been developed, including *in vitro* virus isolation by culture, serologic assays, enzyme-linked immunosorbent assay (ELISA), and polymerase chain reaction (PCR)-based assays. However, various disadvantages make these methods less than ideal in their practical application. For example, *in vitro* virus isolation by culture is time-consuming and requires about 10 days; the criteria for serologic detection of influenza virus, the hemagglutination inhibition (HI) assay, has been proved to have low sensitivity and cannot detect this kind of antibody that senses diverse avian influenza viruses [6,7]; PCR-based assays are more sensitive, but plenty of mismatches between the primers and AIV sequences can happen and those assays cannot distinguish the live viruses from inactivated viruses [8,9]. Moreover, virus isolation, serological methods and PCR-based assays often require highly trained lab workers and time-intensive procedures, as well as a highly sterile experimental environment [9,10].

Aptamers are artificial oligonucleic acid or peptide molecules that can bind to a specific molecule such as amino acids, drugs, viruses, proteins, other molecules and even cells, tissues and organisms with high affinity and selectivity. Aptamers were developed in 1990 at two independent labs: the Gold lab [11] and the Szostak lab [12] in the USA. Recently, a variety of aptasensors were investigated for different applications, such as an electrochemical biosensor for detecting the interferon gamma (IFN-γ) [13], a capacitive biosensor for the detection of C-reactive protein [14], and a fluorescent biosensor for measurement of potassium ion [15]. Some research on aptasensors for the detection of various viruses have been reported, including the HIV-1 Tat protein [16], hepatitis C virus [17], and herpes virus [18]. Some of them chose RNA aptamers which are less stable under harsh conditions and cannot be easily used in the field when compared with DNA aptamers. Very recently, Li *et al.* [19,20] developed aptamers specific to AIV H5N1 using the SELEX method and their results showed the aptamers were compatible with available monoclonal antibodies in both specificity and affinity. SELEX refers to the systematic evolution of ligands by exponential enrichment which begins with a random sequence combinatorial library of oligonucleotides which are screened by a repeated process of *in vitro* selection and amplification [21]. Each member in a library is a linear oligomer of a unique sequence and the molecular diversity is dependent on the number of randomized nucleotide positions [21].

The SPR technology has been in use for almost three decades since 1982 [22]. SPR biosensors have also seen rapid development and improvement since the first use of SPR for biosensing purposes, as an effective alternative for analyzing biological interactions. There are many research papers that have reported SPR biosensors and their applications in protein immobilization [23,24], antibody selection and detection [25,26], bacteria immobilization [27], papillomavirus genotype [28], diagnosis of hepatitis B virus and dengue virus [29,30]. Some researchers have described SPR biosensors for avian influenza DNA hybrization [31], adamantane binding sites in the influenza A M2 ion channel [32], influenza virus hemagglutinin monitoration [33], and binding kinetics study [34], but no report was found on a SPR aptasensor for detection of AIV. In this research, a portable SPR aptasensor was developed for rapid detection of AIV H5N1 based on the specific DNA aptamer selected by our laboratory [20] and the Spreeta sensor manufactured by Texas Instruments (Dallas, TX). The miniature Spreeta SPR sensing chip is a fully intergrated SPR sensor element containing a light emitting diode (LED) light source, a gold SPR surface, a reflecting mirror that directs the reflected light to a photodiode array and a temperature sensor, with size only of 4 cm (length) × 2 cm (height) × 1 cm (width). Following the conjugation of AIV H5N1 specific aptamer to the sensor surface, the aptasensor was ready for H5N1 detection. The total detection time was within 1.5 h, which is faster than all conventional methods for AIV detection, such as virus isolation and identification (5∼7 days), ELISA (3 h) and RT-PCT (3∼5 h).

## 2. Experimental Section

### 2.1. Reagents

**Figure 1.**
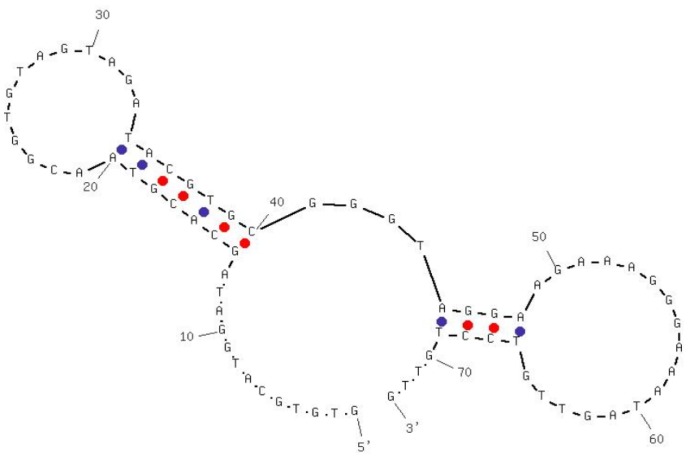
Sequence and predicted secondary structure of the aptamer used in this study.

Aptamers specific against H5N1 were described in detail in our previous study [19,20]. Briefly, selection and characterization of DNA aptamers were carried out using Systematic Evolution of Ligands by EXponential enrichment (SELEX) technology and surface plasmon resonance (SPR). The selection was started with an ssDNA (single-stranded DNA) library of 10^14^ molecules randomized at central 74 nt. For the first four selection cycles, purified hemagglutinin (HA) from AIV H5N1 was used as the target protein, and starting from the fifth cycle, entire H5N1 virus was applied in order to improve the specificity. After thirteen rounds of selection, DNA aptamers that bind to the H5N1 were isolated and three apatmer sequences were further characterized by sequencing and affinity binding. The best aptamer candidate had a dissociation constant (*K_D_*) of 4.65 nM as determined by SPR, showing a strong binding between the HA and the selected aptamer. The specificity was determined by testing non-target AIV H5N2, H5N3, H5N9, H9N2 and H7N2. Negligible cross-reactivity confirmed the high specificity of selected aptamers. The sequence and predicted secondary structure of the aptamer used in this study is shown in Figure 1.

Streptavidin was from Rockland (Gilbertsville, PA, USA). Biotin-aptamers were synthesized by Integrated DNA Technologies (IDT, Coralville, IA, USA). Inactivated AIV H5N1 was provided by USDA-APHIS National Veterinary Services Laboratories (NVSL) in Ames (IA, USA); inactivated non-target AIV H1N1, H2N2, H5N2, H7N2 and H9N2 were from Animal Diagnostic Laboratory (ADL), at Pennsylvania State University (University Park, PA, USA); poultry swab samples were prepared by Poultry Health Laboratory, University of Arkansas (Fayetteville, AR, USA). Phosphate buffered saline (PBS), sodium hydroxide, hydrochloric acid, and ethanol were purchased from Sigma-Aldrich (St. Louis, MO, USA); Millipore water with 18 MΩ·cm of resistivity was obtained with a Milli-Q system (Millipore, Bedford, MA, USA).

### 2.2. Apparatus

The Spreeta SPR detector was purchased from Texas Instruments (Dallas, TX, USA), and the system includes SPR sensor, integrated multichannel flow cell, and 12-bit interface box. A DELL laptop, Latitude D610 was used to collect the data. One mL-syringes were purchased from Sigma-Aldrich (St. Louis, MO, USA); nitrocellulose membrane was bought from Schleicher & Schuell BioScience Inc. (Keene, NH, USA).

### 2.3. Fabrication of the SPR Aptasensor

**Figure 2.**
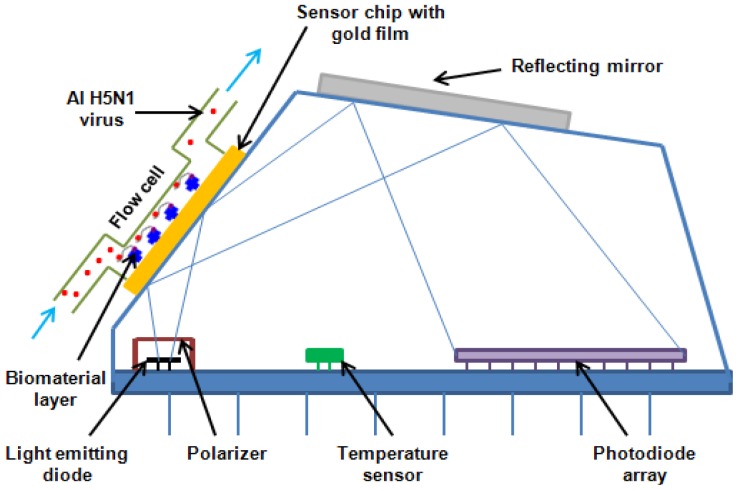
The configuration for measuring AIV using the portable SPR aptasensor.

A surface plasma wave (SPW) is an electromagnetic wave which can be generated at the interface between a dielectric and a metal [35]. The RI of an SPW can be expressed as Sellmeier Equation:

(1) $${RI}^{2}=1+\sum \left[\frac{{\text{B}}_{\text{n}}\lambda^{2}}{\lambda^{2}-{\text{C}}_{\text{n}}}\right]$$

where λ is the vacuum wavelength, B*_n_* and C*_n_* are experimentally determined *Sellmeier coefficients* which can be found in the RefractiveIndex.info database [36]. The SPR technique is applied for measuring the RI of materials immobilized on a metal surface [37]. Figure 2 shows the configuration for measuring AIV using the portable SPR aptasensor.

The miniature Spreeta SPR sensing chip is a fully intergrated SPR sensor element containing an LED (light emitting diode) light source, a gold SPR surface, a reflecting mirror that directs the reflected light to a photodiode array and a temperature sensor. The intensity of polarized light reflected off a thin layer of gold on the surface of a prism shows a dependence on the angle of incidence. Attachment of viruses on the aptamer immobilized gold surface resulted in the change of thickness of the gold surface, and consequently the change of angle. The difference between angles (before and after) is highly related with the properties of the viruses.

### 2.4. SPR Detection of AI Virus

#### 2.4.1. AI Virus in Pure Culture

**Figure 3.**
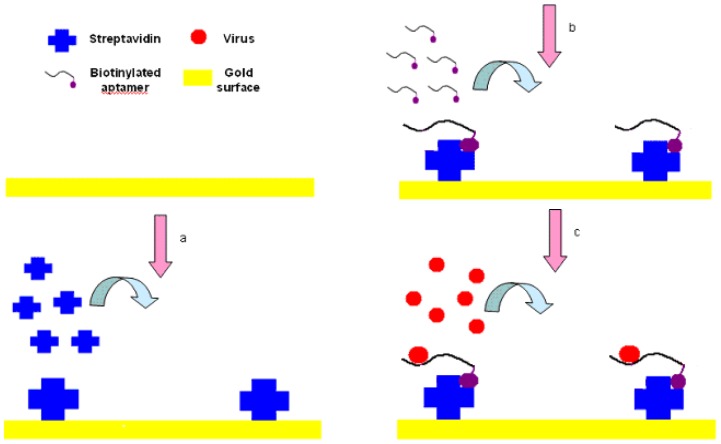
Principle of SPR biosensor for detection of AIV H5N1: (**a**) Streptavidin immobilization; (**b**) Biotinylated aptamer immobilization; (**c**) Virus detection.

The experimental setup is shown in Figure 3. First, streptavidin powder (5 mg) was dissolved with PBS (1 mL, 10 mM, pH 7.4). Then the 5 mg/mL of streptavidin solution was packed into 1.5-mL tube with a 200 μL/tube and stored at −20 °C for future tests. The Au surface of SPR biosensor was pretreated with NaOH (300 μL, 1 M) for 20 min and HCl (300 μL, 1 M) for 5 min in order to get rid of any irregularities and obtain a clean Au surface [37]. After pretreatment, the crystals were rinsed by spraying ethanol and water successively, and dried in a stream of nitrogen.

The SPR sensor was then installed in the flow cell, initialized in the air and calibrated in deionized water. PBS (1 mL, 10 mM, pH 7.4) was injected into the flow cell of the SPR chip, and flowed for 3 to 5 min until the PBS baseline became unchanged. The streptavidin was then injected and immobilized onto the cleaned Au surface through physical adsorption by applying streptavidin (250 μL, 0.2 mg/mL) to the flow cell and incubated for 25 min. After that, the surface was rinsed using an excess volume of PBS (1 mL, 10 mM, pH 7.4) to remove the unbound streptavidin, and get the stabilized RI value for streptavidin within 3 to 5 min. Biotinylated aptamer (300 μL, 2.04 μg/mL) was injected into the flow cell to conjugate to the binding sites on the streptavidin and incubated for 25 min, followed by PBS solution (1 mL, 10 mM, pH 7.4) for 3 to 5 min to rinse off the excess aptamers resulting in the stabilized RI value for aptamers. Finally, AIV H5N1 (300 μL) diluted with PBS (from 0.0128 to 12.8 HAU) was injected into the channel on the Au surface, incubated for 25 min, and then the excess was removed by rinsing with PBS (1 mL, 10 mM, pH 7.4). Then, the stabilized RI value for virus could be obtained within 3 to 5 min. The change in RI value caused by AIV H5N1 was calculated as the difference between the stabilized RI values for virus and aptamers. All tests were repeated three times.

The haemagglutination (HA) is defined as the agglutination of red blood cells which is caused by an antibody or by the existence of viruses or other microbes [38]. One HA unit (HAU) in the haemagglutinin titration is the minimum amount of virus that will cause complete agglutination of the red blood cells [39]. ELD_50_ (50% Egg Lethal Does) is a common concentration units for AIV and 128 HAU/50 μL equals to 1 × 10^6.2^ ELD_50_/mL [40].

#### 2.4.2. AI Virus in Poultry Swab Samples

Poultry swab samples were obtained from the saliva in the chicken throat. Twelve tubes with swabs were prepared. Two birds as well as 1 mL of PBS were used for each tube. First, each tube with swabs was mixed sufficiently. After mixing, each swab was squeezed against the tube wall several times and then discarded. All tubes of the solution, then, were combined into one tube. Finally, the solution was filtered using syringe filter (0.45 μm) and was spiked with AIV H5N1 for further use. The original AIV H5N1 titers were diluted to different concentrations (from 0.128 to 12.8 HAU) in the poultry swab solution. Pure swab solution without spiking with AIV H5N1 was used as a control. The procedure for detection of AIV in poultry swab samples was the same as that for detection of AIV in PBS except the sample that was prepared by mixing AIV H5N1 with poultry swab wash solution instead of PBS. All tests were repeated three times.

## 3. Results and Discussion

### 3.1. Optimization of Parameters with the Reagents

**Figure 4.**
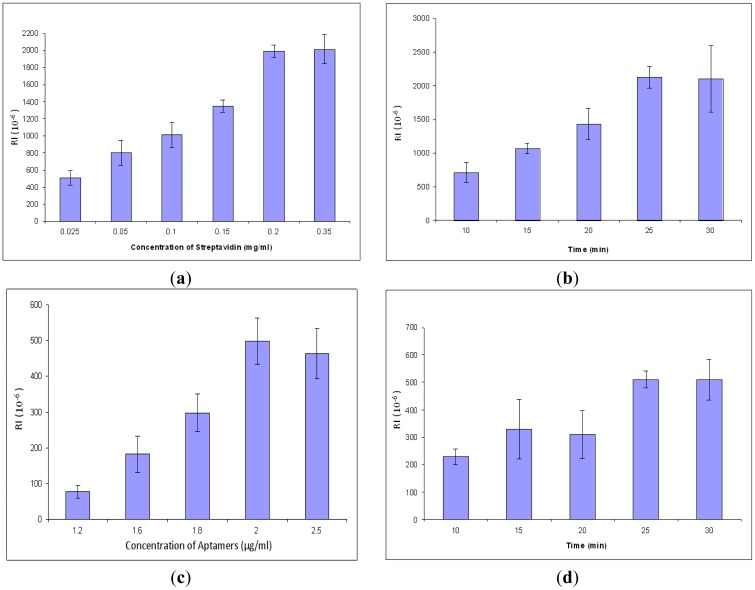
Optimization of streptavidin and aptamers, each concentration and incubation time repeated three times, respectively: (**a**) Streptavidin concentration; (**b**) Streptavidin incubation time; (**c**) Aptamer concentration; (**d**) Aptamer incubation time.

In order to obtain better signals and conserve reagents, the concentration and incubation time of streptavidin and aptamers were optimized. All procedures are the same in the concentration optimization except the concentration of streptavidin. The result for optimization of streptavidin concentration is shown in Figure 4(a). The response values increased gradually from 0.025 to 0.2 mg/mL, but after 0.2 mg/mL, the values became unchanged. The results showed that the optimal concentration for streptavidin for the SPR aptasensor was 0.2 mg/mL. After that, streptavidin incubation time was optimized by varying incubation time at a constant value of concentration. The optimal incubation time for streptavidin was 25 min (Figure 4(b)). Similarly, the parameters of aptamers were optimized, resulting in 2.0 μg/mL of optimized concentration (Figure 4(c)) and 25 min of optimized incubation time (Figure 4(d)).

### 3.2. Detection of AIV H5N1 in PBS

**Figure 5.**
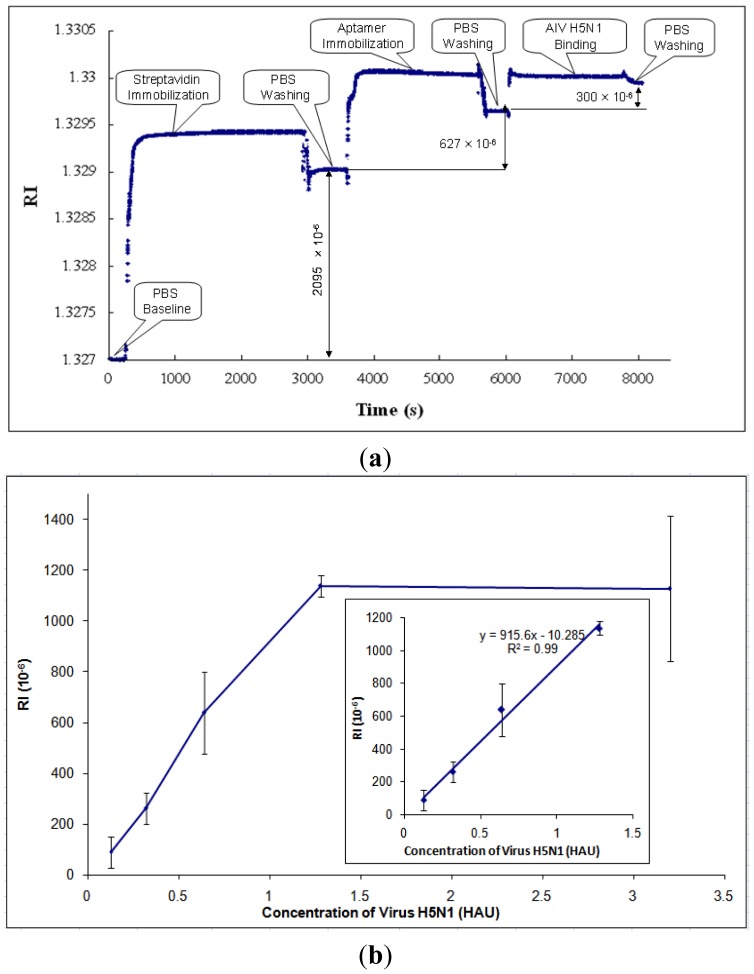
Detection of AIV H5N1 using SPR aptasensor: (**a**) A typical response curve of the SPR aptasensor to the surface modification and AIV H5N1 detection; (**b**) Calibration curve for AIV H5N1 detection (all tests were repeated three times).

After parametric optimization, AIV H5N1 was detected as described in Section 2.4. A typical response curve of AIV H5N1 detection using SPR biosensor is shown in Figure 5(a). The PBS baseline is at the beginning of the curve, RI = 1.327. Then, streptavidin was immobilized onto the gold surface of the SPR chip, causing the RI to increase. In order to rinse off the excess streptavidin, PBS was applied to the chip surface, resulting in the stabilized RI value for streptavidin. The RI change caused by streptavidin was taken as the difference between the PBS baseline and the stabilized RI value for streptavidin (RI = 2,095 × 10^−6^, as shown in Figure 5(a)). Similarly, the RI changes caused by aptamers and AIV H5N1 were obtained by the differences between the stabilized RI values for aptamers and streptavidin, and the stabilized RI values for virus and aptamers, respectively.

Based on the collected data, the RI value showed a great linear correlation (R^2^ of 0.99) with the concentration of AIV in the range from 0.128 to 1.28 HAU (Figure 5(b)). After the concentration of AIV became greater than 1.28 HAU, the signal tended to become constant (Figure 5(b)). The signals for concentrations of AIV of less than 0.128 HAU were not detectable. The value of RI for AIV at 0.125 HAU was more than three times any noises, including non-target AIV subtypes, thus, the detection limit [41] for this biosensor was 0.128 HAU. The calibration curve of H5N1 detection was obtained and is shown in Figure 5(b).

In this research, some non-target AIV subtypes such as H1N1, H2N2, H5N2, H5N9, H7N2, and H9N2 were tested using the developed SPR aptasensor. The experimental procedure was the same as that for the H5N1 target except for the AIV subtypes. Negligible signal (<4% of H5N1) was observed from those non-target AIV subtypes (Table 1). The results indicated that the developed aptasensor has good specificity to the target AIV H5N1, which was comparable with the results obtainable with Dot-blot [20].

Each of the SPR chips was washed with NaOH (300 μL, 1 M) for 20 min and HCl (300 μL, 1 M) for 5 min and reused five to seven times until a 50% decrease of the RI value was observed. The decrease of signal value was probably caused by part of the Au surface peeling off, as observed in the tests.

### 3.3. SPR Biosensor for the Detection of AI H5N1 Virus in Poultry Swab Samples

**Figure 6.**
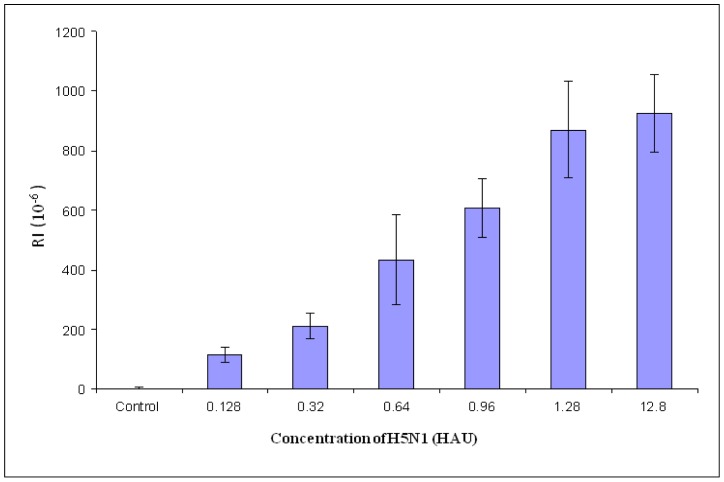
SPR aptasensor for detection of AIV H5N1 in poultry swab samples.

The procedure for detection of poultry swab samples was the same as that for target H5N1. The AIV H5N1 was dissolved with poultry swab sample instead of PBS which was used in the previous detection of the pure virus. The lower detection limit of the poultry swab samples was the same as that of the pure virus, which was 0.128 HAU (Figure 6). With the increase of the virus concentration, the value of RI increased (Figure 6). Due to the impurities and/or other materials like other proteins in the swab samples which might have a negative impact on the binding of virus to the aptamer, the number of virus particle that were responsible for the detection signal may be less than that of virus diluted with PBS. The experimental results showed that the developed biosensor was able to detect AIV H5N1 in poultry swab samples.

### 3.4. Comparison Study

A comparison study between the aptasensor and other methods for AIV H5 subtype detection is summarized in Table 2. It indicated that the developed SPR aptasensor has acceptable sensitivity, and advantages in rapid detection, portability, being label-free and allowing real-time detection. Our on-going research will focus on the application of the SPR aptasensor for detection of AIV H5N1 in the field.

## 4. Conclusions

An SPR aptasensor for the detection of AIV H5N1 in poultry swab samples was designed, fabricated, and tested. The optimum parameters were obtained for the concentration and incubation time of streptavidin (0.2 mg/mL, 25 min) and aptamers (2.0 μg/mL, 25 min). A good correlation (R^2^ = 0.99) was found between AIV concentration in the range of 0.128 to 1.28 HAU and the RI. The specificity of SPR aptasensor was confirmed by comparison of AIV H5N1 with other non-target AIV subtypes such as AIV H1N1, H2N2, H5N2, H7N2, and H9N2, which showed no interference. The developed SPR aptasensor was able to detect AIV H5N1 in poultry swab samples with a lower detection limit of 0.128 HAU within 1.5 h. The SPR aptasensor has the potential to provide the poultry industry with a new and convenient method for in-field detection of avian influenza.

## Figures and Tables

**Table 1.** Specificity study of the developed SPR biosensor.

**Virus Subtype (0.32 HAU)**	**RI (10^−6^)**			**Mean ± SD (RI 10^−6^)**
Control	5	−7	1	0 ± 6
H1N1	−13	2	0	−4 ± 8
H2N2	2	−10	66	−1 ± 8
H5N2	9	−1	22	10 ± 12
H5N9	−4	1	13	3 ± 9
H7N2	19	8	−6	7 ± 13
H9N2	4	1	0	2 ± 2
H5N1	315	196	276	262 ± 61

**Table 2.** A comparison study between the aptasensor and other methods for AIV H5 subtype detection.

**Methods**	**Detection Time**	**Detection Limit**	**Advantages**	**Disadvantages**	**Reference**
Virus isolation and identification	5∼7 days	1 EID_50_/mL	“Gold standard”, sensitive, accurate	Time consuming, complicated operation	[41]
ELISA	3 h	1.0 ng	Rapid	High rate of false positives	[42]
RT-PCR	5 h	0.0256 HAU	Good in specificity and sensitivity	Expensive and highly skilled	[43]
Real time RT-PCR	3 h	10 copies/reaction	Rapid, good in specificity and sensitivity	Expensive and complicated operation	[44]
Nucleic acid sequence-based amplification (NASBA)	6 h	10^1.92^ EID_50_/mL	Good in specificity and sensitivity	High rate of false positives	[45]
SPR Aptasensor	1.5 h	0.128 HAU	Rapid, portable, label free and real-time detection	In-field detection will be needed for the on-going research	This study
